# Incidence, predictors, and management of postoperative subdural empyema following chronic subdural hematoma evacuation: a population-based cohort study

**DOI:** 10.1007/s00701-025-06561-0

**Published:** 2025-05-23

**Authors:** Sophia Jansson, Nike Halvardsson Flores, Ali Buwaider, Helge Johansson, Akhar Shokri Stenström, Jiri Bartek, Alexander Fletcher-Sandersjöö

**Affiliations:** 1 Department of Neurosurgery, Karolinska University Hospital, Stockholm, Sweden; 2 Department of Neurosurgery, Uppsala University Hospital, Uppsala, Sweden; 3 Infectious Diseases Unit, Karolinska University Hospital, Stockholm, Sweden; 4 Department of Clinical Neuroscience, Karolinska Institutet, Stockholm, Sweden

**Keywords:** Burr-hole, Chronic subdural hematoma, Craniotomy, Neurosurgery, Postoperative infection, Subdural empyema

## Abstract

**Purpose:**

Subdural empyema (SDE) is a rare but potentially serious complication following chronic subdural hematoma (CSDH) evacuation. This study aimed to establish the incidence of postoperative SDE, identify risk factors for its development, characterize the bacterial pathogens involved, and evaluate optimal surgical management strategies.

**Methods:**

Patients aged ≥ 15 years who underwent CSDH evacuation at the Karolinska University Hospital between 2006 and 2022 were retrospectively screened for postoperative SDE. Logistic regression analyses were used to identify predictors of SDE development and treatment failure.

**Results:**

Among 2656 operations for CSDH, 37 (1.4%) resulted in postoperative SDE. Independent predictors of SDE were larger CSDH diameter (odds ratio [OR] 1.12, 95% confidence interval [CI] 1.06 – 1.17, *p* < 0.001) and Cloxacillin prophylaxis during index CSDH-surgery (OR 4.63, 95% CI 2.19 – 11.0, *p* < 0.001). Hemiparesis (54%) and wound infection (30%) were the most common SDE symptoms, and fever was frequently absent. Cutibacterium acnes was the most common bacterial isolate, identified in 76% of cases. Craniotomy was more effective than burr-hole evacuation for managing SDE, with the latter showing a higher risk of reoperation (OR 11.5, 95% CI 1.72 – 230, *p = *0.032). The median antibiotic treatment duration was 48 days (interquartile range 35–77). One-year mortality did not differ significantly between patients with and without SDE (8.1% vs. 12%, *p = *0.618).

**Conclusion:**

A larger CSDH diameter and Cloxacillin prophylaxis significantly increased the risk of postoperative SDE. Craniotomy was more effective than burr-hole evacuation for SDE management, and one-year mortality was not elevated in patients who developed an SDE.

## Introduction

Chronic subdural hematoma (CSDH) is among the most common neurosurgical conditions, with an annual incidence ranging from 1.72 to 20.6 per 100,000 individuals [21]. With an aging population, the incidence of CSDH is rising [14, 21], and burr-hole evacuation remains the gold-standard treatment for symptomatic CSDH with significant mass effect. However, several postoperative complications can occur, with subdural empyema (SDE), a purulent collection between the dura mater and the arachnoid, being one of the potentially more serious if left untreated [7].

The risk of developing a postoperative SDE after CSDH evacuation has been reported to range from 0.8% to 2.1% [1, 3, 7, 13, 16, 17] (Table 1). Due to this, fewer than 50 cases have been reported in the literature, leaving a significant gap in our understanding of several critical aspects of this complication. For example, predictors of SDE development have not been identified, and the optimal surgical strategy for managing SDE is also unclear. Additionally, the most common bacterial pathogens have not been identified, which hampers the selection of empirical antibiotic treatment. To address these knowledge gaps, we analyzed a large population-based cohort of patients who underwent CSDH surgery with the aim to:

-
  - Identify predictors of SDE development following CSDH evacuation
  - Identify the most common bacterial pathogens involved
  - Evaluate optimal surgical strategies for SDE evacuation

**Table 1:** Summary of previous cohort studies reporting on SDE following chronic subdural hematoma evacuation

Study	Design	CSDH (n)	SDE (n, %)	Conclusion regarding SDE
Bartek et al. (2017) [1]	Retrospective cohort study	759	7 (0.9%)	Only incidence rate reported
Chan et al. (2017) [3]	Retrospective cohort study	302	3 (1.0%)	Only incidence rate reported
El Ouadih et al. (2020) [7]	Retrospective cohort study	203	2 (1.0%)	Antibiotic prophylaxis did not affect SDE rates
Nayil et al. (2012) [13]	Retrospective cohort study	1181	9 (0.8%)	All SDE patients presented with hemiparesis within three weeks post-CSDH evacuation; all underwent craniotomy; two (22%) died
Rauhala et al. (2020) [16]	Retrospective cohort study	978	20 (2.0%)	SDE was more common in patients who underwent CSDH reoperation (4.3% vs. 0.9%)
Rohde et al. (2002) [17]	Retrospective cohort study	376	8 (2.1%)	Only incidence rate reported

*CSDH* chronic subdural hematoma, *SDE* subdural empyema

By exploring these areas, our study sought to enhance the understanding of this rare but potentially serious complication.

## Methods

### Study design and population

We conducted a single-center retrospective study of all patients aged ≥ 15 years who underwent surgical treatment for a CSDH from 2006 to 2022. Patients were identified using the procedure code"AAD10"(evacuation of CSDH) in the Orbit surgical management system (Evry Healthcare Systems, Solna, Sweden). From this cohort, we systematically screened for patients who developed a postoperative SDE by manually reviewing each patient's medical record in the TakeCare electronic records system (CompuGroup Medical Sweden AB, Farsta, Sweden). Imaging data were retrieved from the Sectra Picture Archiving and Communication System (PACS) IDS7 (Sectra AB, Linköping, Sweden). The study was approved by the regional ethical review board (EPN 2017/247 and EPN 2013/591–31/1), who waived the need for informed consent. We excluded patients with CSDHs in arachnoid cysts, external hydrocephalus, or prior neurosurgery within the prior six months, to align with the methodological standards of randomized controlled trials in the field [2, 8, 15]. We also excluded those who’s initial CSDH evacuation was performed at another hospital, to ensure complete access to perioperative data and standardized treatment protocols.

### Variables

Demographic and comorbidity data included age, sex, diabetes mellitus, moderate-to-severe renal disease (defined as chronic kidney disease stage ≥ 3), and the Charlson Comorbidity Index, which was calculated based on recorded diagnoses at the time of surgery. Clinical and radiological variables included the preoperative Glasgow Coma Scale score, hematoma diameter, midline shift, and whether the CSDH was bilateral. Hematoma diameter was defined as the maximal thickness in the coronal orientation perpendicular to the skull curvature. In cases of bilateral CSDH, the maximal diameter was measured on each side, and the larger of the two was used for analysis. Midline shift was measured at the point of greatest displacement from the midline on the preoperative CT scan. Treatment-related variables included operative time (measured from skin incision to closure), type of anesthesia (local or general), surgical method (burr-hole or mini-craniotomy), type of perioperative antibiotic prophylaxis, and whether a CSDH reoperation was performed within six months of the index procedure.

For patients who developed a postoperative SDE, we collected the time from index surgery to SDE evacuation, presenting symptoms, C-reactive protein (CRP) levels prior to reoperation, and the highest recorded body temperature within 24 h of SDE evacuation. Additional data included empyema diameter and consistency (liquid or semi-solid), the type of surgical procedure used for SDE evacuation (burr-hole or craniotomy), and the bacterial isolates identified from intraoperative cultures. We also recorded the total duration of antibiotic treatment (including intravenous and oral components), whether hyperbaric oxygen therapy was used, whether SDE reoperation was required, and one-year mortality.

### CSDH treatment protocol

All patients were managed under a standardized protocol for their initial CSDH evacuation. CSDHs were radiologically confirmed using computed tomography (CT) or magnetic resonance imaging (MRI), supplemented by perioperative confirmation as needed. A single dose of intravenous antibiotics was administered preoperatively, though precise timing and any potential intraoperative redosing were not consistently documented. From the start of the study until April 2014, the standard prophylaxis consisted of 2 g intravenous Cloxacillin. Beginning in May 2014, this was replaced by 1.5 g intravenous Cefuroxime. For patients unable to tolerate these regimens, 600 mg intravenous Clindamycin was used in both time periods. The standard surgical approach during the entire study period involved a single burr-hole craniostomy under local anesthesia, followed by saline irrigation and a 24-h active subgaleal drainage, defined as a closed-suction drain connected to a compressible bulb reservoir. Irrigation was typically performed using saline at body temperature, except during periods when the center participated in a randomized trial comparing irrigation temperatures [2]. Postoperative CT scans were obtained at the discretion of the treating physician, most commonly in cases of neurological deterioration or if the patient failed to improve clinically. Follow-up was conducted by telephone or in person, typically within 1–2 months postoperatively.

### Definition of postoperative SDE

Diagnosing a postoperative SDE can be challenging because it often resembles recurrent CSDH on CT scans [6, 19], and no standardized definition currently exists [1, 3, 7, 13, 16, 17]. In this study, we defined an SDE as having occurred when the below two criteria were met:

1. Perioperative findings suggestive of infection, including purulent or pus-like fluid, foul odor, thickened or inflamed dura and subdural membranes, or granulation/fibrous tissue in the subdural space.
2. Positive bacterial growth from cultures obtained during SDE evacuation, initiated at the surgeon’s suspicion of infection.

### Statistical analyses

All continuous data deviated from a normal distribution pattern (Shapiro–Wilk test p-value < 0.05); therefore, we presented them as medians with interquartile ranges (IQRs). Categorical data were presented as counts and percentages. Patients were stratified based on the development of SDE, and mortality rates were compared using the Chi-square test. A Kaplan–Meier curve was employed to illustrate the cumulative risk of SDE development over time from index CSDH surgery. We then conducted two separate sets of univariable and step-down multivariable logistic regression analyses. The first aimed to identify predictors of SDE development following index CSDH surgery, and the second focused on determining predictors of reoperation after initial SDE evacuation. Missing data were handled through listwise deletion. All statistical analyses were performed using R version 4.1.2, and a p-value < 0.05 was considered statistically significant.

## Results

### Baseline characteristics

**Fig. 1:**
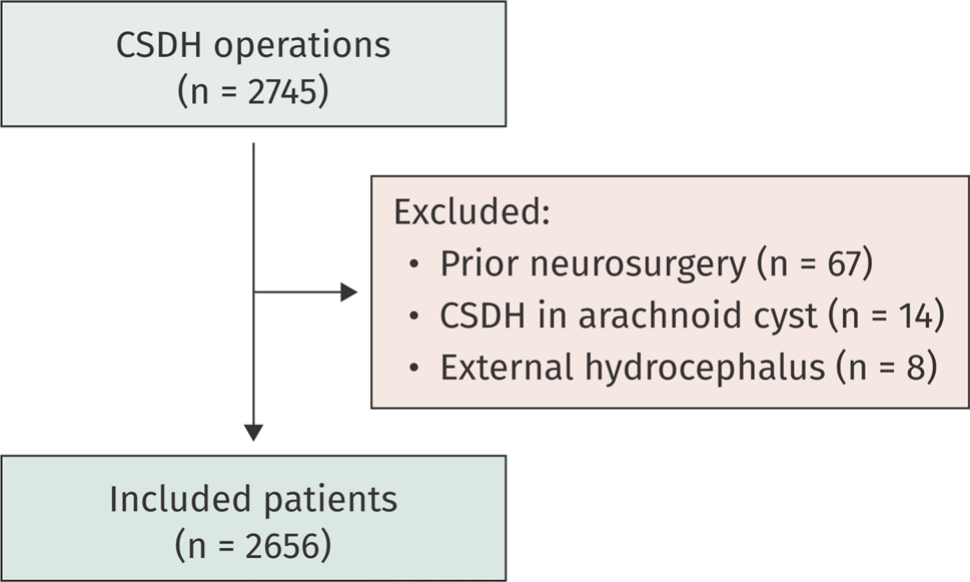
Flow chart of the patient selection process

During the study period, 2745 patients underwent a CSDH evacuation. Of these, 89 were excluded due to prior neurosurgery within 6 months (*n =* 67), CSDH in an arachnoid cyst (*n =* 14) and external hydrocephalus (*n =* 8). The remaining 2656 patients were included in this study (Fig. 1). Their median age was 76 years (IQR, 68–83), and 72% were male. The median Charlson comorbidity index was 1.0 (IQR 0.0–2.0). Most procedures (93%, *n =* 2476) were performed under local anesthesia. Within 6 months of the initial surgery, 10% of patients had required reoperation due to a CSDH recurrence (Table 2).

**Table 2:** Overview of the study cohort

Variable	All patients (*n =* 2656)	Postoperative SDE	
		**No (*n =* 2619)**	**Yes (*n =* 37)**
Age (years)	76 (68–83)	76 (68–83)	75 (66–82)
Male sex	1910 (72%)	1878 (72%)	32 (86%)
Diabetes	430 (16%)	424 (16%)	6 (16%)
Moderate-to-severe renal disease	102 (3.8%)	100 (3.8%)	2 (5.4%)
Charlson comorbidity index	1.0 (0.0–2.0)	1.0 (0.0–2.0)	1.0 (0.0–2.0)
Pre-operative GCS	15 (14–15)	15 (14–15)	15 (14–15)
Hematoma diameter (mm)	21 (17–25)	21 (17–25)	25 (23–27)
Midline shift (mm)	7.0 (4.0–11)	7.0 (4.0–11)	8.0 (5.0–10)
Operative time (skin-to-skin) (min)	29 (20–41)	29 (20–41)	27 (20–37)
Bilateral CSDH evacuation	445 (17%)	440 (17%)	5 (14%)
Local anesthesia only	2476 (93%)	2440 (93%)	36 (97%)
Surgical method			
Burr-hole	2408 (91%)	2374 (91%)	34 (92%)
Mini-craniotomy	248 (9.3%)	245 (9.4%)	3 (8.1%)
CSDH reoperation	266 (10%)	259 (9.9%)	7 (19%)
Days from CSDH surgery to empyema evacuation	-	-	23 (17–39)
1-year mortality	312 (12%)	309 (12%)	3 (8.1%)

Data are presented as median (IQR) or count (%). *CSDH* chronic subdural hematoma, *GCS* Glasgow Coma Scale, *mm* millimeters, *SDE* subdural empyema. Bold *p-*values indicate statistical significance (*p* < 0.05)

### Incidence and predictors of SDE

**Fig. 2:**
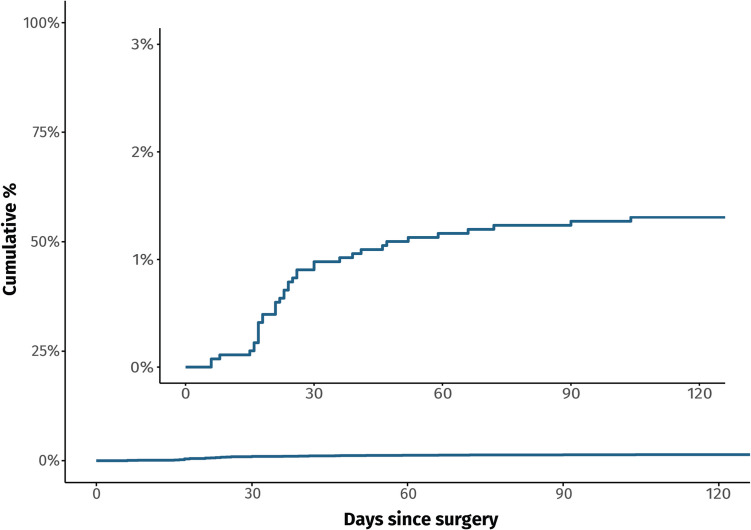
Kaplan–Meier plot illustrating the time from index chronic subdural hematoma evacuation to surgical intervention for postoperative subdural empyema

Among 40 patients reoperated due to suspected infection, 37 (1.4%) had positive intraoperative cultures and were classified as SDE. The median time from initial CSDH surgery to SDE evacuation was 23 days (IQR 17–39, Fig. 2). Initial CSDH diameter (odds ratio [OR] 1.12, 95% confidence interval [CI] 1.06–1.17, *p* < 0.001) and Cloxacillin prophylaxis (OR 4.63, 95% CI 2.19–11.0, *p* < 0.001) were identified as independent predictors of SDE development (Table 3).

**Table 3:** Univariable and multivariable logistic regression predicting empyema development

Variable	Univariable model		Final step-down multivariable model	
	**OR (95% CI)**	***p*-value**	**OR (95% CI)**	***p-*value**
**Demographics**				
Age (years)	0.99 (0.97–1.02)	0.646	–	–
Male sex	2.53 (1.07–7.41)	0.055	–	–
Diabetes	1.00 (0.37–2.25)	0.996	–	–
Moderate-to-severe renal disease	1.44 (0.23–4.81)	0.620	–	–
**Imaging findings**				
Hematoma diameter (mm)	**1.11 (1.05–1.16)**	** < 0.001**	**1.12 (1.06–1.17)**	** < 0.001**
Midline shift (mm)	1.03 (0.96–1.10)	0.462	–	–
**Treatment-related variables**				
Cloxacillin prophylaxis (*ref: Cefuroxime*)	**4.09 (1.95–9.63)**	** < 0.001**	**4.63 (2.19–11.0)**	** < 0.001**
Operative time (min)	1.00 (0.99–1.02)	0.743	–	–
Bilateral CSDH evacuation	0.77 (0.26–1.83)	0.596	–	–
CSDH evacuation using mini-craniotomy	0.85 (0.20–2.40)	0.796	–	–
CSDH reoperation	2.13 (0.85–4.62)	0.076	–	–

*CSDH* chronic subdural hematoma, *mm* millimeters, *min* minutes, *ref* reference. Bold *p-*values indicate statistical significance (*p* < 0.05). Dashes (–) used for excluded variables in the final step-down model

### SDE presentation and bacterial isolates

Among the 37 patients with an SDE, the most common presenting symptom was hemiparesis (55%), followed by wound infection (30%), dysphasia (22%), confusion (14%), headache (14%), and gait instability (8.1%). The median CRP level prior to SDE evacuation was 66 mg/L (IQR 34–131). The most frequently isolated pathogen was Cutibacterium acnes (76%), followed by Staphylococcus aureus (16%), Coagulase-negative staphylococci (5.4%), Klebsiella aerogenes (5.4%), Finegoldia magna (5.4%), and Enterobacter cloacae (2.7%) (Table 4). No patient underwent a preoperative diagnostic puncture; all cultures were collected intraoperatively.

**Table 4:** Clinical presentation and microbiological findings

Variable	SDE patients (*n =* 37)
Empyema diameter (mm)	23 (19–25)
Bilateral empyema	5 (14%)
Presenting symptom	
Hemiparesis	20 (54%)
Wound infection	11 (30%)
Dysphasia	8 (22%)
Confusion	5 (14%)
Headache	5 (14%)
Gait instability	3 (8.1%)
CRP prior to empyema evacuation (mg/L)	66 (34–131)
Highest body temperature within 24 h prior to empyema evacuation, °C	37.2 (36.8–37.9)
Microbiological culture	
Cutibacterium acnes	28 (76%)
Staphylococcus aureus	6 (16%)
Coagulase-Negative Staphylococci	2 (5.4%)
Klebsiella aerogenes	2 (5.4%)
Finegoldia magna	2 (5.4%)
Enterobacter cloacae	1 (2.7%)

Data is shown as median (interquartile range) or count (proportion). *C* Celsius, *CRP* C-reactive protein, *mm* millimeters, *SDE* subdural empyema

### Treatment of SDE and predictors of SDE reoperation

For the SDE surgery, 18 (49%) received a burr-hole evacuation and 19 (51%) underwent a craniotomy. SDE consistency was semi-solid in 25 patients (68%) and liquid in 12 (32%) (Table 5). Empiric antibiotic therapy was initiated in all cases and adjusted based on microbiological findings and clinical response. Further changes were commonly made at the time of oral transition. The median antibiotic treatment duration was 48 days (IQR 35–76), with a median of 11 days (IQR 7.0–29) of intravenous antibiotics and 30 days (IQR 30–56) of oral therapy. Five patients had an antibiotic treatment duration < 4 weeks, due to death (*n =* 3), non-compliance (*n =* 1), and non-tolerable side effects (*n =* 1). The latter two did not require an SDE reoperation. Hyperbaric oxygen therapy was utilized in 5 patients (14%) (Table 5).

**Table 5:** Treatment approaches and outcomes

Variable	SDE patients (*n =* 37)
Surgical method	
Burr-hole evacuation	18 (49%)
Craniotomy	19 (51%)
Empyema consistency	
Liquid	12 (32%)
Semi-solid	25 (68%)
Antibiotic treatment	37 (100%)
Treatment duration (days)	48 (35–76)
Intravenous antibiotics (days)	11 (7.0–29)
Oral antibiotics (days)	30 (9.0–56)
Hyperbaric oxygen chamber	5 (14%)
Empyema reoperation	8 (22%)

Data is shown as median (interquartile range) or count (proportion). *SDE* subdural empyema

Eight patients (22%) required a reoperation for their SDE (Table 5). In the regression analysis predicting SDE reoperation, burr-hole evacuation was independently associated with a higher risk compared to craniotomy (OR 11.5, 95% CI 1.72–230, *p = *0.032). Other factors, such as age, preoperative CRP, time to surgery, specific bacterial pathogens, and SDE consistency, were not linked to reoperation risk (Table 6).

**Table 6:** Univariable and multivariable logistic regression predicting empyema reoperation

Variable	Univariable model		Final step-down multivariable model	
	**OR (95% CI)**	**p-value**	**OR (95% CI)**	***p*-value**
Age (years)	1.04 (0.97–1.07)	0.341	–	–
Pre-operative CRP	0.99 (0.98–1.01)	0.377	–	–
Empyema diameter (mm)	1.01 (0.88–1.16)	0.867	–	–
Cutibacterium acnes	2.67 (0.38–54.0)	0.392	–	–
Staphylococcus aureus	2.08 (0.25–13.7)	0.453	–	–
Burr-hole evacuation	11.5 (1.72–230)	**0.032**	11.5 (1.72–230)	**0.032**
Semi-solid state	0.38 (0.07–1.95)	0.239	–	–

*CRP* C-reactive protein. Bold text in the p-value column indicates a statistically significant correlation (*p* < 0.05). Dashes (–) used for excluded variables in the final step-down model

## Discussion

We conducted the largest population-based study to date examining SDE after CSDH evacuation, aiming to determine its incidence, define predictive factors, identify the main bacterial pathogens, and evaluate optimal surgical strategies.

### Incidence and predictors of SDE

Our analysis revealed a 1.4% incidence of postoperative SDE, aligning with previously reported rates of 0.8–2.1% [1, 3, 7, 13, 16, 17]. Initial CSDH diameter emerged as an independent predictor of SDE development. Possible explanations include a larger subdural space that fosters bacterial growth after CSDH evacuation, longer operative times, and incomplete evacuation of larger CSDH leaving residual blood to fuel infection. In addition, Cloxacillin prophylaxis was linked to a 4.6-fold higher risk of SDE compared to Cefuroxime. This is consistent with a prior study at our center showing that switching from Cloxacillin to Cefuroxime reduced the surgical site infection rate from 13.3% to 5.4% in tumor surgery [18]. The likely explanation is Cloxacillin’s narrower spectrum, primarily targeting staphylococci and streptococci, with insufficient coverage against Cutibacterium acnes, the predominant SDE pathogen in our cohort. Cefuroxime, in contrast, offers broader-spectrum activity and better pharmacokinetics (including enhanced central nervous system penetration) [9]. These findings underscore the importance of tailoring prophylactic antibiotics to local resistance patterns and highlight the need for periodic reassessment of prophylaxis protocols.

Other variables, including age, sex, comorbidities, surgical approach, and previous CSDH reoperation, did not show significant associations with SDE development in our cohort. This finding contrasts with Rauhala et al., who observed higher SDE rates among patients requiring CSDH reoperation [16]. However, the univariable p-value for reoperation in our study was 0.076, suggesting that an association, though not statistically confirmed, cannot be entirely ruled out. Further investigation with larger sample sizes should help clarify this relationship.

### Presenting symptoms and diagnostic challenges

Hemiparesis (55%) was the most common SDE symptom, followed by wound infection (30%) and dysphasia (22%), mirroring the results of Nayil et al. [13]. In all our cases of wound infection, the bacteria isolated matched those identified during SDE evacuation. It should also be noted that 65% of the SDE patients had normal body temperatures, illustrating how SDE can mimic recurrent CSDH both clinically and radiologically. Surgeons must therefore remain alert to the potential of an underlying SDE when encountering postoperative wound complications or unexplained neurological deterioration. MRI, as well as an increased CRP, may offer improved specificity in discriminating between recurrent CSDH and SDE.

### Bacterial pathogens

Cutibacterium acnes was the most frequently isolated organism, followed by Staphylococcus aureus, a pattern suggesting intraoperative contamination as a major mechanism in SDE pathogenesis. Previous reports have similarly identified Cutibacterium acnes [4], Staphylococcus aureus [7], Klebsiella oxytoca [20], and E. coli [5] as SDE pathogens. Notably, 24 of the 28 Cutibacterium acnes cases in our cohort occurred when Cloxacillin was the standard prophylaxis, underscoring its limited anaerobic coverage. Although culture contamination is a known concern with Cutibacterium acnes, we required both positive cultures and perioperative evidence of infection to distinguish pathogenic infection from potential contaminants. Given this, we believe our findings strongly support prophylactic antibiotic regimens with sufficient activity against anaerobes – particularly Cutibacterium acnes – to reduce the risk of postoperative SDE.

### Optimal surgical management

Craniotomy was more effective than burr-hole evacuation for treating SDE, as burr-hole patients had a markedly higher likelihood of SDE reoperation (OR 11.5, 95% CI 1.72–230, *p = *0.032). This observation aligns with prior recommendations [5, 11–13], presumably because craniotomy facilitates more comprehensive removal of infected tissue. Burr-hole evacuation is likely particularly inadequate for semi-solid SDE, which constituted 65% of our cases. Consequently, surgeons opting for burr-hole procedures should be prepared to transition to craniotomy if they encounter poor drainage or partitioned SDE. Anesthesia choice also comes into play here, since craniotomy typically necessitates general anesthesia.

### Postoperative antibiotic strategies and outcome

Every SDE patient in our cohort received antibiotic therapy, which was generally designed to cover the most common pathogens in postoperative SDE, including coagulase-negative staphylococci, Cutibacterium acnes, anaerobes, and gram-negative bacilli. Empiric regimens typically included a third-generation cephalosporin or carbapenem in combination with a glycopeptide or oxazolidinone. Adjustments were often made within the first 24 h after infectious disease consultation, followed by further tailoring based on culture results and again during the transition from intravenous to oral therapy.

The median treatment duration was 48 days (IQR 35–76), generally conforming to guidelines for spontaneous SDE [10]. As most cases were evacuated using burr-hole craniostomy without bone flap fixation devices, the risk of biofilm formation was minimal, explaining why more prolonged courses were not required. We excluded treatment duration from our regression models to avoid reverse causality: reoperations may have led to extended antibiotic courses rather than vice versa. Although the optimal duration remains poorly defined, our data suggest a practical range of 5–10 weeks, guided by clinical response, microbial data, inflammatory marker trends, and imaging evidence of resolution.

Lastly, one-year mortality was similar between those who developed SDE (7.5%) and those who did not (12%, *p = *0.618), suggesting that timely recognition and appropriate management can yield outcomes comparable to patients without this complication.

### Study limitations

This study’s retrospective, single-center design limits the generalizability of our findings, and reliance on medical records restricted the evaluation of long-term functional outcomes. Our definition of SDE – requiring intraoperative findings plus positive cultures – is not standardized, raising the possibility of both missed and overdiagnosed cases. Moreover, because only those who underwent reoperation could be definitively diagnosed, patients who deteriorated rapidly or never returned for surgery may have harbored undetected infections, further leading to an underestimate of the true incidence. We also lacked detailed data on certain patient-specific risk factors (e.g., nutritional status and smoking), which may influence SDE susceptibility. Moreover, we did not include functional or neurological outcomes, as follow-up varies considerably between patients, and including this data would likely introduce selective bias. Looking ahead, prospective, multicenter studies that adopt standardized diagnostic criteria, gather more comprehensive clinical data, and evaluate functional outcomes will be important for addressing these limitations and refining SDE management strategies.

## Conclusion

In this population-based analysis of 2656 CSDH operations, 1.4% of patients developed a postoperative SDE – most often caused by Cutibacterium acnes. A larger baseline CSDH diameter and the use of Cloxacillin rather than Cefuroxime for surgical prophylaxis significantly elevated the risk of SDE. Craniotomy was superior to burr-hole evacuation for managing postoperative SDE, as the latter carried a higher likelihood of reoperation. Despite the clinical burden posed by SDE, one-year mortality rates were similar in patients with and without this complication, underscoring the value of early diagnosis and appropriate treatment.

## Data Availability

The datasets used and/or analyzed during the current study are available from the corresponding author on reasonable request.

## Abbreviations

CI: Confidence interval
CRP: C-reactive protein
CSDH: Chronic subdural hematoma
CT: Computed tomography
IQR: Interquartile range
MRI: Magnetic resonance imaging
OR: Odds ratio
PACS: Picture Archiving and Communication System
SDE: Subdural empyema
